# Supplementary material for: Unbiased K-mer Analysis Reveals Changes in Copy Number of Highly Repetitive Sequences During Maize Domestication and Improvement
Source: Sci Rep. 2017 Feb 10;7:42444. doi: 10.1038/srep42444 (PMC5301235; doi:10.1038/srep42444)
Supplement: Supplementary Information [file srep42444-s1.doc]

Unbiased K-mer Analysis Reveals Changes in Copy Number of Highly Repetitive Sequences During Maize Domestication and Improvement

Sanzhen Liu, Jun Zheng, Pierre Migeon, Jie Ren, Ying Hu, Cheng He, Hongjun Liu, Junjie Fu, Frank F. White, Christopher Toomajian, Guoying Wang

**Table S2**. Statistics of functional classes of HAKmers

| Code | Class | Number of all non-redundant k-mers | Number of differential abundance k-mers | Differential/all (%) |
| --- | --- | --- | --- | --- |
| TERTOOT | Unclassified Retrotransposons | 470,660 | 27 | 0.006 |
| TERT002 | Ty3-gypsy | 21,824 | 0 | 0 |
| OTKN000 | Knob | 10,371 | 8,269 | 79.732 |
| CMCMOOT | Unclassified Centromere Sequences | 9,837 | 81 | 0.823 |
| OTOT000 | Unclassified | 9,330 | 41 | 0.439 |
| RGRR000 | 45S rDNA | 9,173 | 3,867 | 42.156 |
| TERT001 | Ty1-copia | 7,657 | 0 | 0 |
| TETN002 | CACTA, En/Spm | 4,469 | 0 | 0 |
| TETNOOT | Unclassified Transposons | 3,112 | 0 | 0 |
| CMCM002 | Centromeric satellite repeats | 1,838 | 621 | 33.787 |
| RGRR005 | 5S rDNA | 1,397 | 4 | 0.286 |
| TRTM000 | Telomere | 656 | 117 | 17.835 |
| TEMT059 | mPIF | 567 | 0 | 0 |
| CMCM001 | Centromere-specific Retrotransposons | 450 | 0 | 0 |
| TETN003 | Mutator (MULE) | 311 | 0 | 0 |
| TETN001 | Ac/Ds | 227 | 0 | 0 |
| TEMT055 | Heart breaker | 135 | 0 | 0 |
| TEMTOOT | Unclassified MITEs | 134 | 0 | 0 |
| TERT003 | LINE | 127 | 0 | 0 |
| TEMT056 | Frequent Flyer | 33 | 0 | 0 |
| TETN004 | Mariner (MLE) | 27 | 0 | 0 |
| TEMT002 | Stowaway | 14 | 0 | 0 |
| TEMT057 | Heart Healer | 12 | 0 | 0 |
| TEMT006 | Castaway | 7 | 0 | 0 |
| TEMT001 | Tourist | 3 | 0 | 0 |

**Table S3**. Sum of estimated copies of all k-mers in each functional class

| Class | Sum of copies of all k-mers per class^*^ | | Mo17/B73 |
| --- | --- | --- | --- |
|  | B73 | Mo17 |  |
| knob | 1,979,523,103 | 896,095,038 | 0.453 |
| 45S rDNA | 807,833,346 | 1,080,176,065 | 1.337 |
| Ty3-gypsy | 802,093,819 | 813,043,361 | 1.014 |
| Unclassified | 301,005,739 | 310,232,395 | 1.031 |
| Unclassified Centromere Sequences | 289,605,185 | 276,527,056 | 0.955 |
| Ty1-copia | 239,439,384 | 245,138,170 | 1.024 |
| 5S rDNA | 96,397,625 | 120,000,151 | 1.245 |
| Centromeric satellite repeats (CentC) | 90,457,272 | 154,423,006 | 1.707 |
| CACTA, En/Spm | 67,240,372 | 66,214,078 | 0.985 |
| Unclassified Transposons | 65,768,661 | 65,767,164 | 1.0 |
| mPIF | 14,768,671 | 15,618,841 | 1.058 |
| Telomere | 13,554,843 | 10,501,889 | 0.775 |
| Centromere-specific Retrotransposons | 9,128,860 | 8,158,594 | 0.894 |
| Mutator (MULE) | 7,728,574 | 8,316,731 | 1.076 |
| Ac/Ds | 7,479,272 | 8,370,891 | 1.119 |
| LINE | 2,541,319 | 2,837,120 | 1.116 |
| Heart breaker | 2,070,377 | 2,162,051 | 1.044 |
| Unclassified MITEs | 1,912,841 | 1,904,293 | 0.996 |
| Frequent Flyer | 566,569 | 538,890 | 0.951 |
| Mariner (MLE) | 458,570 | 465,303 | 1.015 |
| Stowaway | 436,074 | 614,332 | 1.409 |
| Heart Healer | 411,751 | 515,739 | 1.253 |
| Castaway | 326,505 | 319,793 | 0.979 |
| Tourist | 64,804 | 61,979 | 0.956 |

^*^ k-mer counts were corrected by 36 and 35 which represent the k-mer abundance of single-copy k-mers in B73 and Mo17, respectively.

**Table S4**. Number of HAKmers showing various mapping peaks^*^

| Number of peaks | 0 | 1 | 2 | 3 | 4 |
| --- | --- | --- | --- | --- | --- |
| No. B73-gain HAKmers (%) | 35  (0.3) | 8,499  (74.5) | 2,840  (24.9) | 28  (0.2) | 11  (0.1) |
| No. Mo17-gain HAKmers (%) | 78  (3.0) | 2,198  (83.5) | 290  (11.0) | 66  (2.5) | 1  (0.04) |

^*^ The maximum one peak was considered for each chromosome

**Table S5**. Number of each functional class of B73-gain HAKmers showing various mapping peaks

| Repeat category | | Number of mapping peaks | | | | |
| --- | --- | --- | --- | --- | --- | --- |
| Code | Class | 0 | 1 | 2 | 3 | 4 |
| CMCM002 | Centromeric satellite repeats | 0 | 134 | 10 | 0 | 0 |
| CMCMOOT | Unclassified centromere sequences | 0 | 19 | 24 | 0 | 3 |
| OTKN000 | Knob | 32 | 5660 | 2569 | 8 | 0 |
| RGRR000 | 45S rDNA | 0 | 2205 | 0 | 0 | 0 |
| TERTOOT | Unclassified retrotransposons | 0 | 27 | 0 | 0 | 0 |
| TRTM000 | Telomere | 0 | 40 | 58 | 12 | 5 |

**Table S7**. Number of each functional class of Mo17-gain HAKmers showing various mapping peaks

| Repeat category | | Number of mapping peaks | | | | |
| --- | --- | --- | --- | --- | --- | --- |
| Code | Class | 0 | 1 | 2 | 3 | 4 |
| CMCM002 | Centromeric satellite repeats | 1 | 267 | 165 | 43 | 1 |
| CMCMOOT | Unclassified Centromere Sequences | 0 | 0 | 28 | 7 | 0 |
| OTOT000 | Unclassified | 0 | 40 | 1 | 0 | 0 |
| RGRR000 | 45S rDNA | 0 | 1645 | 17 | 0 | 0 |
| RGRR005 | 5S rDNA | 0 | 4 | 0 | 0 | 0 |
| TRTM000 | Telomere | 0 | 0 | 2 | 0 | 0 |

**Table S8**. K-mer abundance of three pairs of k-mers harboring a SNV at 26S rRNA

| Pair | K-mers* | Abundance (% of total of a pair) in B73 | Abundance (% of total of a pair) in Mo17 | Type |
| --- | --- | --- | --- | --- |
| 1 | GG**A**ATTCGGTCCTCCGGATTTTCAA | 82,924 (72%) | 40 (0%) | B73gain |
| 1 | GG**T**ATTCGGTCCTCCGGATTTTCAA | 32,602 (28%) | 152,178 (100%) | Mo17gain |
| 2 | CTTGAAAATCCGGAGGACCGAAT**T**C | 82,900 (72%) | 40 (0%) | B73gain |
| 2 | CTTGAAAATCCGGAGGACCGAAT**A**C | 32,628 (28%) | 152,071 (100%) | Mo17gain |
| 3 | **A**ATTCGGTCCTCCGGATTTTCAAGG | 83,074 (72%) | 40 (0%) | B73gain |
| 3 | CCTTGAAAATCCGGAGGACCGAAT**A** | 32,750 (28%) | 152,247 (100%) | Mo17gain |

* the polymorphic site was highlighted in red and the k-mers of the third pair were reversely complemented.

**Figure S1**. Genome-wide distribution of B73- and Mo17-gain rDNA k-mers.

B73- and Mo17-gain rDNA k-mers that can be perfectly aligned to the reference genome (B73Ref3). Alignment numbers per bin (100 kb) were plotted versus bin physical locations at the B73Ref3. The 10 minimum alignment hits per bin were required for each circle/triangle points.


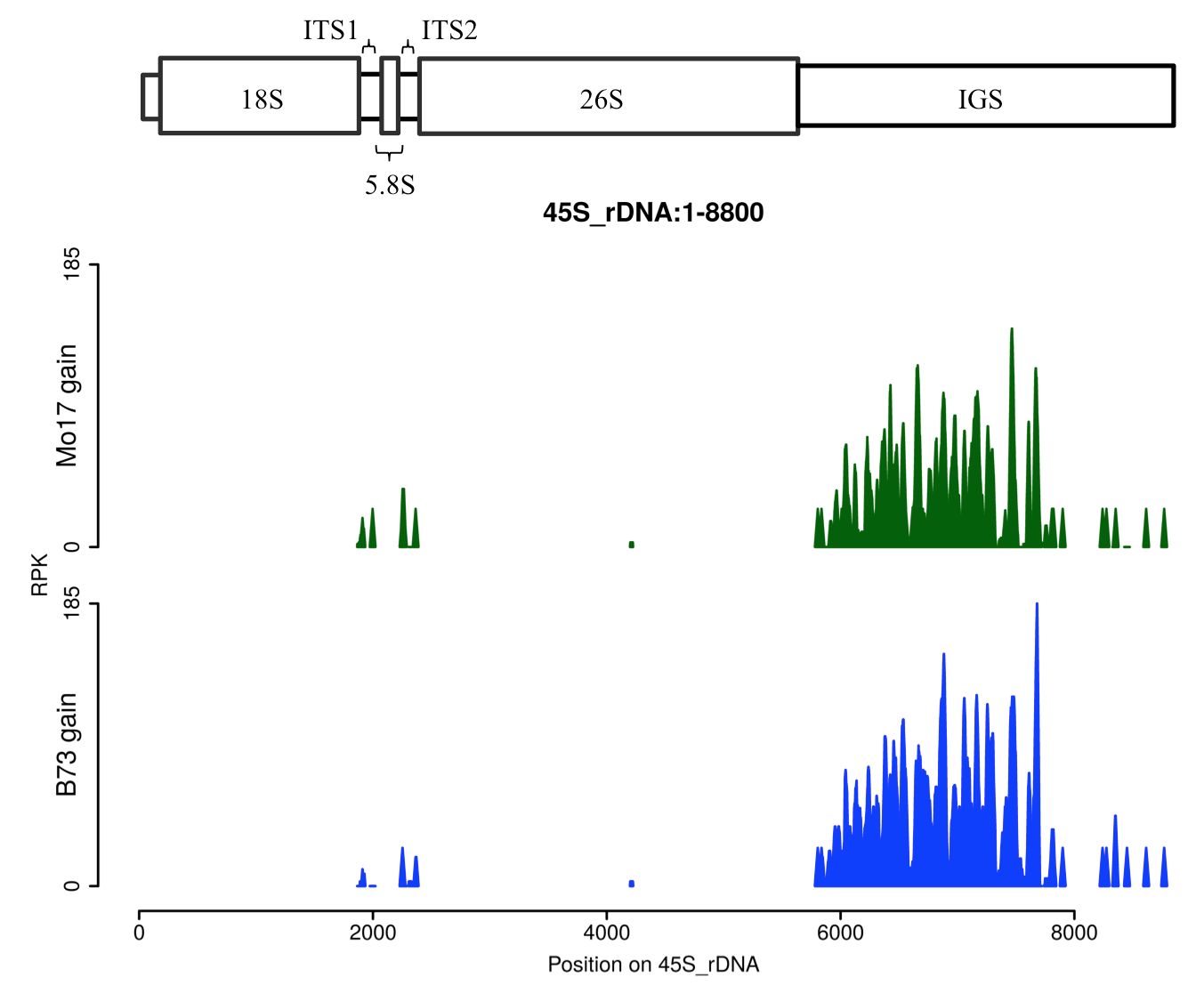


**Figure S2**. Distribution of differential abundance rDNA k-mers on 45S rDNA.

Differential abundance rDNA k-mers, including B73-gain (blue) and Mo17-gain (green), were aligned to the 45S rDNA sequence. The count per 1,000 k-mer alignments (RPK) at each position was plotted versus the position on the 45S rDNA. On the top of the figure, the model structure of 45S rDNA was depicted. Three genes, 18S rRNA, 5.8S rRNA, and 26S rRNA, are included in a 45S rDNA unit. ITS and IGS designate internal transcribed spacer and intergenic spacer, respectively.

**Figure S3**. Barplot of total abundance of B73- and Mo17-specific k-mers.

The total abundance of B73- and Mo17-specific k-mers was determined, normalized, and plotted for each IBM DH line. Bars were colored coded by which genotype of unique k-mers is predominant in that DH line.

**Figure S4. Change patterns of k-mer abundance**

K-mers with significantly differential abundance in teosine, landrace, and improved maize were clustered, resulting in 12 clusters. Each grey line in the figures represents a k-mer. Colored lines are average values from all the k-mers in each cluster. Clusters with a similar pattern were highlighted by the same color.
